# Converging Evidence for Epistasis between *ANK3* and Potassium Channel Gene *KCNQ2* in Bipolar Disorder

**DOI:** 10.3389/fgene.2013.00087

**Published:** 2013-05-17

**Authors:** Jennifer Toolan Judy, Fayaz Seifuddin, Mehdi Pirooznia, Pamela Belmonte Mahon, The Bipolar Genome Study Consortium, Dubravka Jancic, Fernando S. Goes, Thomas Schulze, Sven Cichon, Markus Noethen, Marcella Rietschel, J. Raymond DePaulo, James B. Potash, Peter P. Zandi

**Affiliations:** ^1^Department of Psychiatry, Johns Hopkins School of MedicineBaltimore, MD, USA; ^2^Department of Psychiatry, University of Iowa Carver College of MedicineIowa City, IA, USA; ^3^Genetic Basis of Mood and Anxiety Disorders Unit, National Institute of Mental Health Intramural Research Program, National Institutes of Health, U.S. Department of Health and Human ServicesBethesda, MD, USA; ^4^Department of Genetic Epidemiology in Psychiatry, Central Institute of Mental Health, Mannheim, University of HeidelbergHeidelberg, Germany; ^5^Department of Psychiatry, University of BonnBonn, Germany; ^6^Department of Mental Health, Bloomberg School of Public Health, Johns Hopkins UniversityBaltimore, MD, USA

**Keywords:** epistasis, interaction, bipolar disorder, *ANK3*, *KCNQ2*, channelopathy, ion channel

## Abstract

Genome-wide association studies (GWAS) have implicated *ANK3* as a susceptibility gene for bipolar disorder (BP). We examined whether epistasis with *ANK3* may contribute to the “missing heritability” in BP. We first identified via the STRING database 14 genes encoding proteins with prior biological evidence that they interact molecularly with ANK3. We then tested for statistical evidence of interactions between SNPs in these genes in association with BP in a discovery GWAS dataset and two replication GWAS datasets. The most significant interaction in the discovery GWAS was between SNPs in *ANK3* and *KCNQ2* (*p* = 3.18 × 10^−8^). A total of 31 pair-wise interactions involving combinations between two SNPs from *KCNQ2* and 16 different SNPs in *ANK3* were significant after permutation. Of these, 28 pair-wise interactions were significant in the first replication GWAS. None were significant in the second replication GWAS, but the two SNPs from *KCNQ2* were found to significantly interact with five other SNPs in *ANK3*, suggesting possible allelic heterogeneity. KCNQ2 forms homo- and hetero-meric complexes with KCNQ3 that constitute voltage-gated potassium channels in neurons. ANK3 is an adaptor protein that, through its interaction with KCNQ2 and KCNQ3, directs the localization of this channel in the axon initial segment (AIS). At the AIS, the KCNQ2/3 complex gives rise to the M-current, which stabilizes the neuronal resting potential and inhibits repetitive firing of action potentials. Thus, these channels act as “dampening” components and prevent neuronal hyperactivity. The interactions between *ANK3* and *KCNQ2* merit further investigation, and if confirmed, may motivate a new line of research into a novel therapeutic target for BP.

## Introduction

Genome-wide association studies (GWAS) offer an unbiased, high-throughput approach for searching the entire genome to identify disease-causing variants. This approach has generated much enthusiasm in the study of complex disorders such as bipolar disorder (BP) that have been more difficult than Mendelian diseases to genetically map with linkage and candidate gene association approaches. However, there was little agreement in the findings from the initial GWAS of BP (Craddock and Sklar, 2009). These initial studies were likely under-powered to identify susceptibility loci for BP. A mega-analysis (Ferreira et al., 2008) addressed the issue of power by combining several GWAS, and reported the strongest evidence of an association with *ANK3* (rs10994336, *p* = 9.1 × 10^−9^). A subsequent community-wide effort to combine GWAS from existing studies of BP, referred to as the Psychiatric GWAS Consortium (PGC), identified several additional susceptibility loci and provided further evidence for the association with *ANK3* (rs10994397, *p* = 7.1 × 10^−9^) (Psychiatric GWAS Consortium Bipolar Disorder Working Group, 2011). Although promising, the loci identified thus far from these efforts likely account for only a small proportion of variation in susceptibility. Consequently, much of the genetic architecture of this disorder remains to be elucidated.

The identification of loci that only account for a small proportion of susceptibility to complex disorders appears to be a typical feature of GWAS of complex disorders. This has led investigators to question where the missing heritability is (Manolio et al., 2009). Epistasis, or gene–gene interaction, is one possible explanation for the so-called missing heritability. Epistasis can be conceptualized biologically or statistically (Moore and Williams, 2005). Biologically, it results from physical interactions between DNA, RNA, or proteins such that the resulting phenotype depends on the action of multiple genes. Statistically, it can be defined as a departure from the additive effects of alleles from different loci with respect to their contribution to variation in the resulting phenotype. Searching only for main effects of loci and ignoring possible epistasis may reduce power to detect potentially causal variants (Cordell, 2002). In fact, some researchers have argued that the lack of replication across studies may be a hallmark of epistasis (Wade, 2001; Hirschhorn et al., 2002; Moore and Williams, 2002, 2005). Moreover, the detection of epistasis may help reveal potentially meaningful biological mechanisms (Risch, 1990), which is a central goal of translational research.

The availability of dense SNP maps in GWAS presents the opportunity to investigate potential epistasis across the entire genome. However, this is limited by the computational challenge of testing all possible interactions between SNPs, and the enormous statistical penalty incurred by the multiple testing (Pattin and Moore, 2008). One strategy for overcoming these limitations is to condition the search for epistasis on known loci that have been previously associated with disease. Although this strategy runs the risk of missing purely epistatic models in which the genes involved have little or no main effect on disease susceptibility, it greatly reduces the possible search space, rendering the analyses more efficient, while still exploiting the vast amount of data available from the GWAS. A complementary approach to further reduce the search space involves limiting the analysis to genes whose products are expected to interact based on biological knowledge, such as protein interaction databases (Emily et al., 2009).

We therefore decided to search for evidence of epistasis in BP by focusing on *ANK3*. We chose this gene based on the prior evidence of association with BP, as well as the biological role of its protein product, which lends itself to this type of analysis. ANK3 belongs to a family of multifunctional membrane adapter proteins that target structurally diverse proteins to specialized membrane domains by linking them to the spectrin-based membrane skeleton (Bennett and Chen, 2001). We used a novel strategy that combined bioinformatics and statistical genetics approaches to test for relevant interactions with *ANK3*. First, we searched a well-curated protein–protein interaction database to identify genes encoding proteins that biological evidence suggests interact molecularly with *ANK3*. Then, we tested for congruent statistical evidence of interactions between *ANK3* and these genes using GWAS data. By placing statistical findings in the context of human biology, we sought to strengthen the validity of any conclusions and facilitate the translation of results into potential benefits aimed at prevention and treatment of the disorder (Pattin and Moore, 2008).

## Materials and Methods

### Samples

#### Discovery

We tested for statistical evidence of interactions with *ANK3* using a GWAS dataset that consisted of two samples collected by the NIMH Genetics Initiative Bipolar Disorder Consortium, the Genetic Association Information Network Bipolar Disorder (GAIN) sample, and the Translational Genomics Research Institute Bipolar Disorder (TGEN) sample. The methods for collecting, diagnosing, and genotyping these samples have been described elsewhere (Dick et al., 2003; Kassem et al., 2006; Smith et al., 2009). Cases were ascertained from twelve clinical sites across the United States. They were assessed with the Diagnostic Interview for Genetic Studies (DIGS) (Nurnberger et al., 1994), and family informant data and medical records were obtained. Diagnoses were assigned following a best-estimate procedure according to DSM-III-R or DSM-IV criteria. The cases were all Caucasian and had a diagnosis of BP. Controls were ascertained through the efforts of the Molecular Genetics of Schizophrenia II (MGS-2) Collaboration (Sanders et al., 2008). Control subjects completed a brief psychiatric questionnaire and were excluded if they endorsed a history of BP, psychosis, or major depression. Cases and controls were matched on ethnicity, age, and sex. Appropriate IRB approval was obtained at each collaborating institution, and all subjects provided informed consent.

The cases and controls from both the GAIN and TGEN samples were genotyped on the Affymetrix 6.0 array. Quality control in both samples consisted of dropping subjects with ≥5% missing data, and dropping SNPs with ≥5% missing data, <1% minor allele frequency, or HWE *p*-value <10^−6^ among controls. We then imputed SNPs in each sample and combined them into one dataset. We used phased haplotype data from HapMap I and II release 24^1^ as the reference panel. We used the program BEAGLE to flip orientation to the positive strand and impute estimated allelic dosages for autosomal SNPs (Browning and Yu, 2009). We excluded any SNPs with an imputation *r*^2^ < 0.3. The final GAIN-TGEN GWAS imputed dataset included 2,191 cases and 1,434 controls with data on 3,849,034 total SNPs.

#### Replication

We used two additional GWAS datasets for replication of the top findings. These datasets included the Wellcome Trust Case-Control Consortium (WTCCC) bipolar disorder sample and a German bipolar disorder sample. Methods for collecting, diagnosing, and genotyping both these samples have been described elsewhere (Fangerau et al., 2004; Wellcome Trust Case Control Consortium, 2007; McMahon et al., 2010). Appropriate IRB approval was obtained and all subjects provided informed consent. In the WTCCC sample, 2,000 cases were ascertained from sites across the United Kingdom, and assessed with semi-structured lifetime diagnostic psychiatric interviews (usually the Schedule for Clinical Assessment in Neuropsychiatry). A diagnosis of BP was assigned according to Research Diagnostic Criteria. Three thousand controls were obtained from the 1958 British Cohort study and UK blood donors. All cases and controls were Caucasian. Genotyping was done on the Affymetrix 500K Mapping Array. Quality control consisted of dropping subjects with ≥5% missing data, and dropping SNPs with ≥5% missing data rate, <1% minor allele frequency, <90% quality score, or HWE *p*-value <10^−6^ among controls. After imputation using procedures identical to those used with the GAIN-TGEN dataset as described above, there was a total of 1,868 BP cases and 2,996 controls with data on 3,849,034 SNPs in the final imputed WTCCC dataset.

Bipolar disorder cases for the German sample were recruited through hospital admissions and assessed with a structured interview. Diagnoses were assigned according to a best-estimate procedure using DSM-IV criteria. Population-based control subjects were obtained from the PopGen^2^, KORA^3^, and Heinz Nixdorf Recall Study^4^ cohorts. Genotyping was done on the Illumina HumanHap550 array. Quality control for this dataset included dropping subjects with >5% missing data, and dropping SNPs with >2% missing data, minor allele frequency <2% or HWE *p*-value <0.0001. After imputation using procedures identical to those used with the GAIN-TGEN dataset as described above, there was a total of 645 BP cases and 1,310 controls with data on 3,849,034 SNPs in the final imputed German dataset.

### Bioinformatics resource

We used the STRING database version 9.0^5^ to identify proteins with biological evidence for interaction with *ANK3* (Jensen et al., 2009). STRING is a meta-resource of protein–protein interaction databases, based on the union of known physical protein interactions and curated data from biological pathways. This comprehensive tool extracts evidence from MINT, HPRD, BIND, DIP, BioGRID, KEGG, Reactome, IntAct, EcoCyc, NCI-Nature Pathway Interaction Database and Gene Ontology (GO) protein complexes. It also collects evidence of protein interactions via text mining (from SGD, OMIM, The Interactive Fly, and all abstracts on PubMed) and interaction transfers between organisms, whereby a pair of interacting proteins found in one organism is predicted to occur in another organism if the conservation between the two organisms implies that such a move is justified. Additionally, STRING supplements these known interactions with a computational prediction algorithm. All interactions are given a confidence score according to the joint probabilities from the different lines of evidence, correcting for randomly observed interactions. These scores represent an approximation of how likely each association describes a functional relationship between the two proteins that is at least as specific as that between any given pair of proteins that are annotated in the same KEGG pathway (Szklarczyk et al., 2011).

### Analyses

We adopted an analytic strategy that utilized both bioinformatics and statistical methods to identify and validate significant gene–gene interactions with *ANK3* in BP cases. First, we queried the STRING protein–protein interaction database using ANK3 as the index to identify all proteins that had biological evidence of interaction with it with at least a high confidence score (defined by STRING to be 0.7000 or greater). Then, we analyzed the GAIN-TGEN dataset for statistical evidence of gene–gene interactions associated with BP between *ANK3* and genes encoding the proteins identified by STRING. We used logistic regression to test all possible pair-wise interactions between SNPs in *ANK3* and SNPs in each of the STRING-identified genes, with the gene boundaries defined according to the most inclusive RefSeq transcript (hg18) ±10 kb to include potential regulatory regions. The logistic regression models included terms for each SNP entered as allelic dosages representing the estimated number of copies of the minor allele and an interaction term between the two SNPs. The models also included terms to control for study site and the first two principal components (PCs) from a principal components analysis (PCA) using Eigenstrat (Price et al., 2006) with the genome-wide SNP data to assess for population structure among the samples in the dataset. We obtained the Wald statistic for the interaction terms, and then used permutation procedures to evaluate the significance of the interactions. We randomly permuted the case-control labels in the discovery sample to generate 1,000 replicates. We re-ran the SNP–SNP interaction tests in each replicate, and counted how many of the replicates contained SNP–SNP interactions between *ANK3* and the STRING-identified genes that reached a higher level of significance than what was observed in the original dataset. The total number of replicates out of 1,000 provided an empirical estimate of the significance of the identified interactions while accounting for multiple testing.

We attempted to replicate any SNP pairs that passed the permuted *p*-value threshold, based on the 50th most significant of the 1,000 permuted *p*-values (permuted *p*-value of 0.05). We used the same logistic regression approach in the analysis of the replication samples as was used in the discovery sample.

## Results

A query of the STRING bioinformatics database yielded a total of 16 known proteins that had strong biological evidence (confidence score > 0.7000) for interacting with ANK3. Two of these genes (*L1CAM* and *DMD*) are located on chromosome *X* and were therefore excluded from the analysis, leaving 14 genes of interest (shown in Table 1). We then analyzed the GAIN-TGEN dataset to assess the statistical evidence for interactions between *ANK3* and genes encoding each of the 14 identified proteins in association with BP (Table 2). *KCNQ2* was the most significantly interacting gene, with a minimum *p*-value of 3.18 × 10^−8^, making it the only gene to remain significant after the permutations (permuted *p*-value = 0.005). In total, 31 SNP pairs between *KCNQ2* and *ANK3* were significant after permutation.

**Table 1:** ***ANK3*-interacting genes identified by STRING, ranked by confidence score**.

Gene	Score	Chr	Description
ANK3	–	10	Membrane-cytoskeleton linker. Thought to localize ion channels and cell adhesion molecules at the NoR and AIS
NFASC	0.984	1	Cell adhesion, ankyrin-binding protein. May be involved in neurite extension, axonal guidance, synaptogenesis, myelination, and neuron-glial interactions
SPTBN4	0.964	19	Participates in the clustering of voltage-gated Na(+) channels and cell-adhesion molecules at the NoR and AIS
SCN2A	0.941	2	Sodium channel, voltage-gated, type II, alpha subunit: mediates the voltage-dependent sodium ion permeability of excitable membranes
COL17A1	0.857	10	Collagen, type XVII, alpha 1: may play a role in the integrity of hemidesmosome and the attachment of keratinocytes to the underlying membrane
ARHGEF7	0.833	13	RAC1 guanine nucleotide exchange factor and can induce membrane ruffling. May function in cell migration and as a positive regulator of apoptosis
CACNA1C	0.819	12	Mediates the entry of calcium ions into excitable cells. Involved in a muscle contraction, hormone or neurotransmitter release, gene expression, cell motility, cell division, and cell death
NRCAM	0.766	7	Cell adhesion, ankyrin-binding protein involved in neuron-neuron adhesion. May play a role in the molecular assembly of the NoR
KCNQ2	0.754	20	Probably important in the regulation of neuronal excitability. Joins with KCNQ3 to form a potassium channel with M-current channel properties, which determines the subthreshold electrical excitability of neurons as well as the responsiveness to synaptic inputs
SPTA1	0.735	1	Major constituent of the cytoskeletal network, forms the cytoskeletal superstructure of the erythrocyte plasma membrane
SCN5A	0.726	3	See SNC2A description
KCNQ3	0.72	8	See KCNQ2 description
SPTB	0.719	14	See SPTA1 description
SCN8A	0.716	12	Mediates the voltage-dependent sodium ion permeability of excitable membranes
FADD	0.716	11	Apoptotic adaptor molecule, recruits caspase-8 or -10 to the activated Fas or TNFR-1 receptors

*NoR, nodes of ranvier; *AIS, axon initial segment.

**Table 2:** **Gene-level minimum *p*-values for interactions with ANK3: results from discovery analysis of GAIN-TGEN sample**.

*ANK3* interacting genes	Top SNP pairs (*ANK3*: interacting gene)	Observed *p*-value	Permuted *p*-value
*KCNQ2*	rs1459730: rs2297385	3.18E−08	0.005
*SPTB*	rs12411730: rs2269304	1.18E−05	0.851
*KCNQ3*	rs10994284: rs16904603	1.86E−05	0.939
*NFASC*	rs7098008: rs7535098	1.94E−05	0.949
*CACNA1C*	rs1551684: rs10848666	2.51E−05	0.969
*SCN8A*	rs12355908: rs7976351	9.37E−05	0.992
*SPTBN4*	rs10994198: rs7252109	9.51E−05	0.992
*SCN2A*	rs7901951: rs1439805	9.76E−05	0.992
*ARHGEF7*	rs12355908: rs7993510	0.000107	0.992
*SCN5A*	rs16915451: rs7645173	0.000124	0.992
*NRCAM*	rs16914794: rs13223414	0.00013	0.992
*SPTA1*	rs12356776: rs12039268	0.000157	0.992
*FADD*	rs10821672: rs11235564	0.000283	0.992
*COL17A1*	rs2288359: rs586550	0.00074	0.992

**Figure 1:**
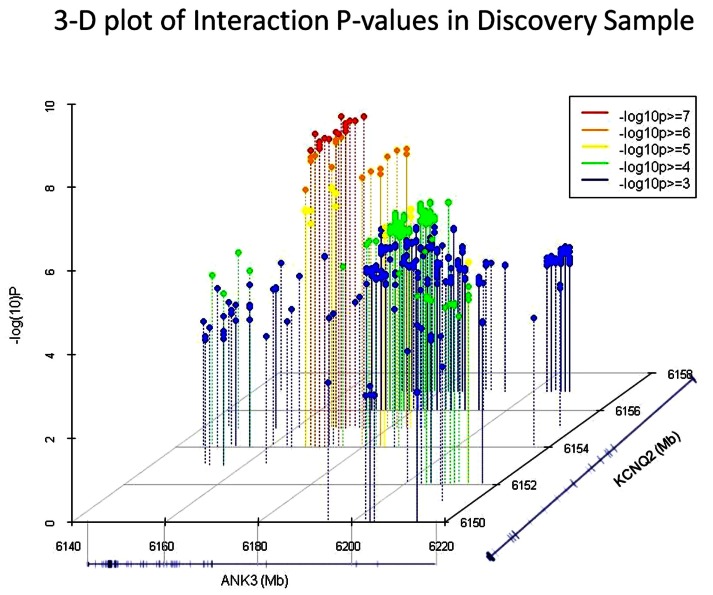
**3D plots of the most significant *p*-values (−log_10_*p* > 3) in the GAIN-TGEN discovery sample**. Of these, the 31 SNP pairs which were significant after permutation correction are shown in red and orange dots. RefSeq gene models representing *ANK3* and *KCNQ2* are shown on the *X* and *Z* axis, respectively, and the −log_10_ (*p*-value) of the interaction tests are shown on the *Y* axis.

Interestingly, the most significant SNP pairs clustered within a distinct peak (Figure 1), and were driven by two SNPs in *KCNQ2*: rs2282150 (an intronic SNP) and rs2297385 (a synonymous coding SNP), both of which are in perfect LD (*r*^2^ = D′ = 1.0) with each other. These two SNPs interacted with 16 unique *ANK3* SNPs that were all intronic (rs1459730, rs16914687, rs898329, rs11814450, rs12413770, rs7911285, rs17233373, rs7910984, rs7093272, rs16914683, rs12413099, rs16914663, rs16914670, rs12416179, rs16914968, and rs10994334). However, a cluster of these *ANK3* SNPs fell within the boundaries of ankyrin repeat domains of the protein, which are known to mediate protein–protein interactions.

Motivated by these results, we sought to replicate the interactions between the 31 SNP pairs in two additional GWAS datasets. Of these 31 significant SNP pairs, 28 were significant (*p* = 0.015–0.048) in the WTCCC sample. The remaining three were suggestive (*p* = 0.052–0.078). None of these SNP pairs were significant in the German dataset. However, interestingly, the two driving SNPs from *KCNQ2* were both involved in significant interactions with five other SNPs in *ANK3* (*p* = 0.033–0.039), suggesting the possibility of allelic heterogeneity. One of these *ANK3* SNPs (rs10994406) is just over 10 kb away from the ANK3 SNP (rs10994397) implicated in the recently reported PGC mega-analysis of BP (Psychiatric GWAS Consortium Bipolar Disorder Working Group, 2011), and the other four (rs10994180, rs10740006, rs10821668, and rs10994181) are tightly clustered in a highly conserved region stretching over 9.5 kb (chr10:61,827,709–61,837,348) with an average phyloP of 1.37 and encompassing an unusually large exon (7,811 bp) in one of the ANK3 transcripts (refseq: NM_020987.3). Two of the SNPs are coding (rs10821668 is a mis-sense and rs10740006 is a synonymous SNP) and the four closely straddle a DNAse hypersensitive site that harbors a transcription factor binding region.

## Discussion

Several GWAS of BP have been reported, but conclusive findings have been elusive. Among the most compelling findings to emerge thus far have implicated the gene *ANK3* (Ferreira et al., 2008; Kelsoe, 2009; Sklar et al., 2011). However, given the observed effect size on risk of this gene, it likely accounts for only a small portion of BP heritability. Because of its known role in interacting with other membrane bound proteins, we examined whether epistasis with *ANK3* might further contribute to susceptibility for BP. We found compelling biological evidence for interactions between *ANK3* and *KCNQ2* based on a bioinformatics search using STRING, and observed congruent statistical evidence of interactions between these genes in association with BP using GWAS data.

**Figure 2:**
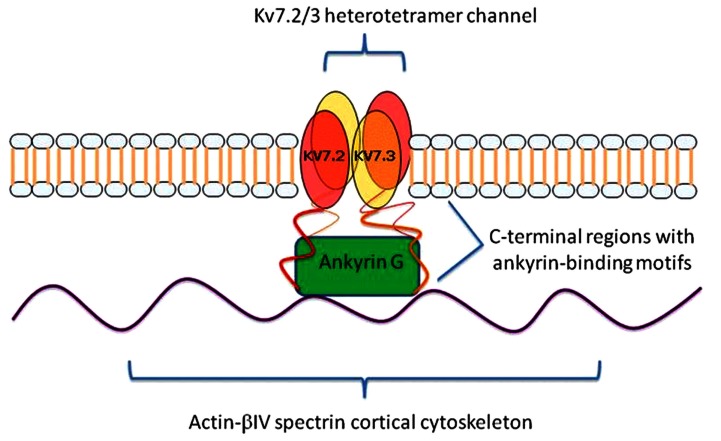
***KCNQ2/3* gene products (Kv7.2 and Kv7.3) form a heterotetramer channel at the axon initial segments (AISs) and nodes of Ranvier of neurons**. Ankyrin-G (*ANK3* gene product) links Kv7.2 and Kv7.3 to the underlying actin-βIV spectrin cortical cytoskeleton and mediates their co-localization via ankyrin-binding motifs at their C-terminal regions.

Prior research has demonstrated an intriguing biological relationship between ANK3 and KCNQ2. ANK3 is a member of the ankyrin family of proteins that link integral membrane proteins to the underlying spectrin-actin cytoskeleton. It is expressed in the central and peripheral nervous system, and helps to regulate the distribution of voltage-gated ion channels (Garrido et al., 2003). In line with this role, ANK3 interacts directly with KCNQ2, which is expressed in the brain and forms homomeric and heteromeric voltage-gated potassium channels with another member of the KCNQ family, KCNQ3. These channels are referred to as Kv7.2/Kv7.3. Through this interaction, ANK3 directs the proper localization of the Kv7.2/Kv7.3 channels to the axonal initial segment (AIS) of neurons and nodes of Ranvier (Figure 2). Pan et al. (2006) demonstrated that the concentration of Kv7.2/Kv7.3 at the AIS was abolished in *ANK3* knock-out mice, and that a short motif in the C-terminal, common to both Kv7.2 and Kv7.3, was found to be responsible for mediating *in vivo* interactions with ANK3 and retention of the subunits at the AIS. At the AIS, Kv7.2/Kv7.3 gives rise to the M-current, which serves to stabilize the neuronal resting potential and inhibit repetitive firing of action potentials. By doing so, these channels act as “dampening” components and prevent neuronal hyperactivity (Delmas and Brown, 2005). Interestingly, mutations in *KCNQ2* have been shown to cause benign familial neonatal convulsions (BFNC), a rare autosomal dominant inherited form of epilepsy (Singh et al., 1998).

Several converging lines of evidence suggest that Kv7.2/Kv7.3 channels and their interactions with ANK3 may play a role in the etio-pathogenesis of BP. Increased excitability of neurons, which is suppressed by the Kv7.2/Kv7.3 channel M-currents, may be characteristic of manic and/or hypomanic periods of BP (Xie and Hagan, 1998). In line with this, one study showed that the suppression of the M-current by *KCNQ2* dominant-negative mutations in a transgenic mouse model led to hyperexcitability of neurons and morphological changes of the hippocampus, and mediated a notable decline in cognitive function, and behavioral changes corresponding to hyperactivity (Peters et al., 2005). Lithium, the leading mood stabilizer, is thought to work via two mechanisms of action that may also influence the activity of Kv7.2/Kv7.3 channels. Lithium inhibits several enzymes involved in inositol recycling, a crucial step in the synthesis of PIP2, a membrane phospholipid that is essential for Kv7.2 activity and for its regulation by acetylcholine (Brown and Passmore, 2009). It also inhibits GSK3β which is responsible for the phosphorylation of Kv7.2/Kv7.3, and phosphorylation of these channels suppresses the M-currents to promote neuronal hyperexcitability (Borsotto et al., 2007). Interestingly, ezogabine, which is a recently approved antiepileptic drug (AED) that targets Kv7.2/Kv7.3 channels, has been shown to have mood stabilizing properties in animal models (Dencker et al., 2008; Redrobe and Nielsen, 2009; Dencker and Husum, 2010; Kristensen et al., 2012) and in a small pilot study in humans (Amann et al., 2006). Finally, a previous genetic study found evidence of an association between variants in *KCNQ2* and BP (Borsotto et al., 2007), and our group has reported genome-wide significant linkage of BP with chromosome 8q24 in a region that harbors *KCNQ3* (Avramopoulos et al., 2004).

Despite the biological and statistical evidence reported here in support of a role for interactions between *ANK3* and *KCNQ2* in the etio-pathogenesis of BP, there are several limitations of the study that merit consideration. Foremost among these is the fact that the same interacting SNP pairs in *ANK3* and *KCNQ2* were not implicated in the German replication dataset. It is important to remember that the SNPs included in these tests are likely not causal and may only partially tag the actual causal variants responsible for disrupting the gene–gene interactions. As a result, different tagging SNPs may be implicated in the interaction tests in different datasets. Moreover, it is quite possible that there is allelic heterogeneity and different causal variants may contribute to the disruption of the gene–gene interactions in different samples. To this point, it is interesting to note that of the *ANK3* SNPs implicated in the German dataset, one was located near the SNP that was most strongly implicated in the PGC mega-analysis of BP, and the other four clustered in another highly interesting region of *ANK3* that may also have important functional relevance that needs to be further elucidated.

Another limitation is that we tested for interactions between SNPs in *KCNQ2* and the top SNPs in *ANK3* implicated in the GWAS by Ferreira et al. (rs10994336) (Ferreira et al., 2008) and in the mega-analysis by the PGC (rs10994397) (Psychiatric GWAS Consortium Bipolar Disorder Working Group, 2011), but none of these were notably significant (data not shown). However, as noted above, a SNP located near the PGC SNP was implicated in one of our replication datasets. It is also likely that the previously identified SNPs in *ANK3* merely tag the true causally related variants and are imperfect proxies for them. The causally related variants in these genes need to be identified before we can more accurately characterize the nature of their interaction in susceptibility to BP. Toward this end, we are currently participating in a sequencing study of BP and plan to examine the data from this study in order to further examine these interactions. By identifying the causally related variants, will be able to examine in *in vivo* and *in vitro* models the mechanisms by which they disrupt the interactions between *ANK3* and *KCNQ2* and contribute to the molecular underpinnings of BP.

As far as we are aware, this is the first comprehensive effort to search for evidence of gene–gene interactions with *ANK3* using genome-wide association data coupled with bioinformatics tools. We have found intriguing evidence that interactions of *ANK3* with *KCNQ2* may contribute to susceptibility for BP. These findings warrant further investigation to identify the causally related variants in these genes and characterize how interactions between them might contribute to the etiology of BP. This may be approached through various avenues, including further genetics studies (interaction analyses using sequencing or linkage data), expression studies (transcriptome or proteomics analyses), or epigenetic studies (methylation or histone modification analyses). These results may be further probed experimentally via animal models. If the interactions of *ANK3* with *KCNQ2* can be confirmed to play a role in the etio-pathogenesis of BP, it would motivate a new avenue of research into a novel therapeutic target for this disease. This would be advantageous because KCNQ2/KCNQ3 channels have been the focus of extensive pharmacologic research in relation to epilepsy, and several compounds that act either as activators or inhibitors of the complex have already been identified and are in various stages of clinical investigation (Miceli et al., 2008).

## Conflict of Interest Statement

The authors declare that the research was conducted in the absence of any commercial or financial relationships that could be construed as a potential conflict of interest.

## Footnotes

^1^http://hapmap.ncbi.nlm.nih.gov/

^2^www.popgen.de

^3^www.gsf.de/KORA

^4^www.recall-studie.uni-essen.de

^5^http://string-db.org/

## Appendix

**Table A1:** **Genotype frequencies by affection status across samples**.

Gene	SNP	Genotype	GAIN-TGEN		WTCCC		German	
			Controls	Cases	Controls	Cases	Controls	Cases
*KCNQ2*	rs2297385	0	0.8601	0.8632	0.8752	0.8683	0.8550	0.8682
		1	0.1329	0.1323	0.1212	0.1290	0.1374	0.1271
		2	0.0070	0.0045	0.0037	0.0027	0.0076	0.0047
*KCNQ2*	rs2282150	0	0.8601	0.8627	0.8755	0.8688	0.8580	0.8698
		1	0.1329	0.1327	0.1208	0.1285	0.1351	0.1256
		2	0.0070	0.0045	0.0037	0.0027	0.0069	0.0047
*ANK3*	rs10994334	0	0.7189	0.7264	0.7336	0.7238	0.7473	0.7566
		1	0.2637	0.2532	0.2480	0.2554	0.2290	0.2248
		2	0.0174	0.0205	0.0184	0.0209	0.0237	0.0186
*ANK3*	rs11814450	0	0.7481	0.7459	0.7553	0.7602	0.7710	0.7736
		1	0.2366	0.2382	0.2293	0.2232	0.2107	0.2047
		2	0.0153	0.0159	0.0154	0.0166	0.0183	0.0217
*ANK3*	rs12413099	0	0.7550	0.7495	0.7630	0.7677	0.7870	0.7891
		1	0.2303	0.2336	0.2226	0.2173	0.1969	0.1922
		2	0.0146	0.0168	0.0144	0.0150	0.0160	0.0186
*ANK3*	rs12413770	0	0.7495	0.7491	0.7640	0.7650	0.7878	0.7907
		1	0.2366	0.2355	0.2216	0.2195	0.1969	0.1907
		2	0.0139	0.0155	0.0144	0.0155	0.0153	0.0186
*ANK3*	rs12416179	0	0.7189	0.7264	0.7336	0.7238	0.7473	0.7566
		1	0.2630	0.2532	0.2480	0.2554	0.2290	0.2248
		2	0.0181	0.0205	0.0184	0.0209	0.0237	0.0186
*ANK3*	rs1459730	0	0.7467	0.7464	0.7593	0.7618	0.7779	0.7814
		1	0.2366	0.2368	0.2260	0.2216	0.2046	0.2000
		2	0.0167	0.0168	0.0147	0.0166	0.0176	0.0186
*ANK3*	rs16914663	0	0.7543	0.7536	0.7627	0.7671	0.7870	0.7891
		1	0.2310	0.2300	0.2230	0.2179	0.1969	0.1922
		2	0.0146	0.0164	0.0144	0.0150	0.0160	0.0186
*ANK3*	rs16914670	0	0.7557	0.7550	0.7634	0.7677	0.7870	0.7891
		1	0.2296	0.2286	0.2223	0.2173	0.1969	0.1922
		2	0.0146	0.0164	0.0144	0.0150	0.0160	0.0186
*ANK3*	rs16914683	0	0.7557	0.7541	0.7634	0.7677	0.7870	0.7891
		1	0.2296	0.2300	0.2223	0.2173	0.1969	0.1922
		2	0.0146	0.0159	0.0144	0.0150	0.0160	0.0186
*ANK3*	rs16914687	0	0.7543	0.7491	0.7627	0.7677	0.7870	0.7891
		1	0.2303	0.2345	0.2230	0.2173	0.1969	0.1922
		2	0.0153	0.0164	0.0144	0.0150	0.0160	0.0186
*ANK3*	rs16914968	0	0.7196	0.7241	0.7350	0.7238	0.7534	0.7581
		1	0.2624	0.2559	0.2470	0.2543	0.2237	0.2248
		2	0.0181	0.0200	0.0180	0.0219	0.0229	0.0171
*ANK3*	rs17233373	0	0.7495	0.7477	0.7640	0.7650	0.7870	0.7891
		1	0.2366	0.2368	0.2216	0.2195	0.1977	0.1922
		2	0.0139	0.0155	0.0144	0.0155	0.0153	0.0186
*ANK3*	rs7093272	0	0.7537	0.7545	0.7630	0.7677	0.7870	0.7891
		1	0.2310	0.2286	0.2226	0.2173	0.1969	0.1922
		2	0.0153	0.0168	0.0144	0.0150	0.0160	0.0186
*ANK3*	rs7910984	0	0.7481	0.7468	0.7510	0.7570	0.7870	0.7891
		1	0.2380	0.2377	0.2343	0.2270	0.1977	0.1922
		2	0.0139	0.0155	0.0147	0.0161	0.0153	0.0186
*ANK3*	rs7911285	0	0.7495	0.7477	0.7640	0.7650	0.7870	0.7891
		1	0.2366	0.2368	0.2216	0.2195	0.1977	0.1922
		2	0.0139	0.0155	0.0144	0.0155	0.0153	0.0186
*ANK3*	rs898329	0	0.7495	0.7491	0.7644	0.7650	0.7878	0.7907
		1	0.2366	0.2355	0.2213	0.2195	0.1969	0.1907
		2	0.0139	0.0155	0.0144	0.0155	0.0153	0.0186
*ANK3**	rs10994180	0	0.5477	0.5382	0.5270	0.5316	0.5328	0.5116
		1	0.3855	0.4009	0.4276	0.4202	0.3939	0.4202
		2	0.0668	0.0609	0.0454	0.0482	0.0733	0.0682
*ANK3**	rs10740006	0	0.5484	0.5391	0.5274	0.5316	0.5328	0.5116
		1	0.3848	0.4000	0.4272	0.4202	0.3939	0.4202
		2	0.0668	0.0609	0.0454	0.0482	0.0733	0.0682
*ANK3**	rs10821668	0	0.5470	0.5395	0.5277	0.5316	0.5328	0.5116
		1	0.3862	0.3995	0.4269	0.4202	0.3939	0.4202
		2	0.0668	0.0609	0.0454	0.0482	0.0733	0.0682
*ANK3**	rs10994181	0	0.5470	0.5386	0.5270	0.5310	0.5321	0.5116
		1	0.3862	0.4000	0.4276	0.4208	0.3947	0.4202
		2	0.0668	0.0614	0.0454	0.0482	0.0733	0.0682
*ANK3**	rs10994406	0	0.9812	0.9818	0.9913	0.9904	0.9511	0.9442
		1	0.0188	0.0173	0.0083	0.0096	0.0481	0.0558
		2	0.0000	0.0009	0.0003	0.0000	0.0008	0.0000

**These five *ANK3* SNPs significantly interact with the driving *KCNQ2* SNPs in the German sample*.

*Genotype frequencies by affection status across the three samples for the two *KCNQ2* SNPs and the 16 *ANK3* SNPs, combinations of which constitute the 28 significant pair-wise interactions in the GAIN-TGEN discovery and WTCCC replication samples. Additionally listed are the five *ANK3* SNPs that interact with the two “driving” *KCNQ2* SNPs that were found to be significant in the German sample*.
